# Development and clinical validation of passive shoulder exoskeleton with novel gravity compensation mechanism for stabilizing arm tremor of surgeons during minimally invasive surgery

**DOI:** 10.3389/fbioe.2024.1418148

**Published:** 2024-12-16

**Authors:** Ho Seon Choi, Seung Jun Lee, Hyunki In

**Affiliations:** ^1^ Department of Artificial Intelligence and Robotics, Sejong University, Seoul, Republic of Korea; ^2^ Center for Healthcare Robotics, Korea Institute of Science and Technology, Seoul, Republic of Korea

**Keywords:** shoulder exoskeleton, minimally invasive surgery, gravity compensation, muscle cocontraction, passive exoskeleton

## Abstract

**Introduction:**

During tasks like minimally invasive surgery (MIS), various factors can make working environment not be ergonomic, and those situations will accumulate fatigue in the surgeon's muscles which will inevitably lead to poor surgical performance. Therefore, there has been a need for technical solutions to solve this problem and one of the methods is exoskeleton robots.

**Methods:**

We designed a passive shoulder exoskeleton whose workspace could be used for MIS to assist the surgeon's movements and performed computational and clinical validation. First, the joint order of the shoulder exoskeleton, which consists of three degrees of freedom, was configured differently from previous studies so that the singularity can be located outside the workspace. And a novel gravity compensation mechanism was developed to replace the existing one, which could no longer be used due to these changes on order of joints. Afterwards, it was computationally verified using statics and kinematics whether sufficient shoulder muscle assistance could be implemented for the entire developed system. Lastly, we manufactured an apparatus that simulated the surgical environment in which the shoulder exoskeleton robot would actually be used, recruited human participants, and conducted an experiment.

**Results:**

Through computational validation, we can guess that the developed shoulder exoskeleton can provide 18.14% reduction of muscle activation to the wearers in workspace. And the results of clinical experiments with human subjects show that activation of deltoid posterior, medial and anterior decreased with average −8.33%, −14.55%, and −21.0%, respectively during MIS-simulated tasks with developed shoulder exoskeleton than without it. And arm tremor which is equals to movement variability also decreased with average 9.85% by using shoulder exoskeleton and maximum −19.5% in a certain position.

**Discussion:**

These experimental results show that our shoulder exoskeleton and its novel gravity compensation mechanism has enough clinical effectiveness for workers of underhead tasks, especially surgeons who conduct MIS. It reduced deltoid activations of wearers and also stabilized arm tremor which are directly related to performance of fine manipulative task, so that this research implies that shoulder exoskeletons are also need for underhead tasks and our shoulder exoskeleton has possibility to contribute to those utilities.

## 1 Introduction

During surgery, the environment in which surgeons operate may become non-ergonomic due to various factors, and an inappropriate work environment causes fatigue in their muscles (Matern, 2009). In the case of interventions in the body cavities, minimally invasive surgery (MIS) method is usually performed to minimize unnecessary side effects on the patient, and due to the nature of this surgical method, the surgeons’ movements are inevitably uncomfortable and limited (Matern et al., 2001; Van Veelen et al., 2002). MIS is a surgery that is performed by making a small incision only in the surgical area with a scalpel and inserting sugical instruments or tools and camera into the hole to prevent contamination of organs which are not targets to be operated (Fuchs, 2002). Surgeons perform surgery by manipulating tools while looking at a sugical monitor in real time. Compared to open surgery, this situation inevitably limits the movement of the doctors’ upper limbs. First of all, the tool is restricted in a small incision and cannot move freely, and because the doctor’s arm joint (Pro/Supination) and the rotation axis of the tool do not match, it is not easy to control the tip of the tool as desired. Because the arm is a multi-joint system, the tool can be moved as intended using the wrist, elbow, and especially the shoulder joints. But unnecessary shoulder movements become too much to compensate for the misalignment of the tool’s axis and the human body’s joints (Perez-Duarte et al., 2013). Due to the characteristics of the surgery, it takes a long time to maintain one posture, and if abduction of the shoulder joint continues, it will cause great fatigue to the three deltoids and problems are bound to occur (Chaffin, 1973; Kaur and Mishra, 2008).

The accumulated fatigue of deltoids makes it difficult for surgeons to control their upper extremity system as desired, which is directly related to the deterioration in surgical performance (Gates and Dingwell, 2011). The performance of surgery depends on how well the surgeon can control the movements of the hand which is the end-point of the patient’s arm system. Human must be able to move their hands accurately and stably to be able to perform tasks that require precision, such as suturing. However, shoulder fatigue makes precise control of this end-point difficult (Blasier et al., 1995; Voight et al., 1996). Usually, in order to perform fine manipulative tasks, a strategy to increase the stiffness of the upper limb joints by increasing the degree of cocontraction of related muscles must be implemented (Fu and Wang, 2009). However, when fatigue accumulates in the muscles, the central nervous system adopts a strategy of lowering the proportion of cocontraction despite of enough energy in order to prevent muscle damage and maintain energy reserves on certain level in the body (Missenard et al., 2008). In addition, even if the joint stiffness is high, accumulated fatigue worsens proprioception, making smooth arm control impossible (Choi and In, 2022). Therefore, as fatigue accumulates, precise control of the arm becomes impossible depending on neuro-physiological factors, and the accumulated fatigue of deltoids inevitably reduces the performance of surgery. In the case of surgical operations, the surgery usually takes several hours, and since this phenomenon is inevitable, ergonomic solutions have been required to solve the problem (Szeto et al., 2012).

Researchers have proposed several methods to try to reduce the load on surgeons’ muscles by changing the surgical environment to be more ergonomic. They usually focus on changing the environment, such as allowing the surgeon to create a comfortable environment by changing the height of the operating table in real time (Manasnayakorn et al., 2009), or adjusting the hand grip of the tool to the axis of the human joint as much as possible (Matern et al., 2002). However, changing the height of the table in real time has the potential to act as a dangerous element in an environment where surgery is performed by making holes in the patient’s body. Also, in some cases, this solution has limitations because the height of the table may not be adjustable at all depending on the patient’s surgical site and hole location. And even if the hand grip is adjusted to be as ergonomic as possible, it is impossible to perfectly match the axis of rotation of the tool with the axis of the human joint, and since the rotation of the tool is affected by where the hole is created, this cannot be a fundamental solution. Therefore, a plan must be prepared to support the strength of the shoulder joint in any situation and position during the surgery.

Among the technologies that are independent of the work environment and can assist the movement of human joints, one of the methods that can be used to solve this problem is an exoskeleton robot system (Gopura et al., 2011). An exoskeleton robot is a type of wearable robot and is developed to be worn by workers and has an actuator or spring to provide power to assist joint movement (Matthew et al., 2015). Actuators can be used to provide customized assistance for various movements, or spring-based gravity compensation mechanisms can be used to decrease the load caused by gravity on human body segments. According to this method, the exoskeleton robot can fundamentally prevent the accumulation of muscle fatigue by assisting the worker’s muscle strength in any posture regardless of the type of tool used or the position of the table. This can also be used to reduce the accumulation of fatigue in deltoids in the shoulder joint.

Normally, the shoulder exoskeleton robots that supports deltoids were developed for workers who typically perform overhead tasks repeatedly for long periods of time (Maurice et al., 2019). Workers working in places such as factory production lines always perform tasks such as assembling or manipulating objects or controllers that are higher than their heads, so shoulder elevation or abduction is bound to last for a long time. Therefore, fatigue accumulates in the deltoids, leading to a decrease in work performance and the occurrence of diseases in workers. To prevent this, shoulder exoskeleton robots have already been commercialized and used (PAEXO and Levitate AIRFRAME) (Latella et al., 2021; Airframe, 2019). Shoulder exoskeleton robots developed for this purpose usually have a gravity compensation mechanism using a spring. Since it always supports the load on the arm due to gravity in the opposite direction, it can reduce the degree of fatigue accumulated even if shoulder elevation continues for a long time. Although there are cases where these robots have been experimentally applied in surgical environments (Liu et al., 2018), those studies only expressed the effectiveness of exoskeleton robots as scores through user surveys and did not analyze them with quantitative indicators such as reduction in muscle activation or stabilization of movements. Perhaps because these shoulder exoskeleton robots were originally developed with a focus on the overhead workspace, quantitative analysis or measurements may show that they are not optimal when used on workers performing tasks with a workspace located below the heads.

Since the shoulder joint has 3° of freedom (McCausland et al., 2023), when manufacturing an exoskeleton robot, three orthogonal joints must exist in succession, which inevitably results in singularity so that this can cause problems when using an exoskeleton robot with a different workspace considered. The shoulder exoskeleton robot to assist overhead tasks has a base joint that is responsible for abd/adduction (Airframe, 2019), which is rotation of the shoulder joint within the transverse plane. The next joint connected to the base joint and responsible for arm elevation exists with a gravity compensation mechanism, and is designed to provide assistance when lifting the arm at any rotation angle from the base joint. And finally, using the semi-cylindrical shaped arm-robot interface, the wearer can freely implement pro/supination of the arm. In this structure, in the standing posture with the arms down, the base joint and the joint responsible for the last pro/supination overlap, putting it in a state of singularity. In fact, the location of this singularity is not a problem when performing work in an overhead workspace, but when used by a surgeon performing MIS, this neutral posture occupies a long period of time during the surgery, and therefore a situation in which the singularity point will be passed during the surgery may occur (Szeto et al., 2012; Aitchison et al., 2016). In this case, the surgeon’s arm may not be able to get out of the singular point, or the robot may be twisted and receive abnormal assistive force, which can lead to a dangerous situation that can affect the surgery.

For this reason, in order for an exoskeleton robot to be used in MIS, it is necessary to design the system considering its workspace. Since the posture of the upper arm coming down in the direction of gravity is included in the workspace, the position of the base joint must be changed to use it in MIS. The options for changing the base joint are the remaining two joints, but since the pro/supination joint that is in direct contact with the arm cannot be selected, so it must be replaced with the joint responsible for abd/adduction (Manasnayakorn et al., 2009). In the case of the gravity compensation mechanism currently in use, the horizontal rotation joint, which is not affected by changes in the size of gravity even when rotated, is the base, so shoulder elevation can assist the movement in any direction. However, if this base joint is changed to another one, the existing gravity compensation mechanism cannot be used. There are also shoulder exoskeleton robots that do not use this type of gravity compensation mechanism and reduce the load through an instrument located between the arm and the side torso which name is PAEXO (Latella et al., 2021). Using this type of robot can avoid singular position within workspace, however, the interface between the arm and the robot must be composed of a strap or velcro, and the pro/supination of the arm cannot be implemented properly. This problem not only impairs the performance of fine manipulative tasks, but because the strap is used, the volume of the muscle that expands when the arm muscle contracts cannot be properly allowed, which inevitably causes discomfort in wearing and has a negative effect on muscle activation. And since the arm is supported from below, there is a structure between the arm and the torso, which interferes arm movement with manipulative tasks like surgery. These robots are also developed considering overhead workspace, so there are problems in applying them to MIS. Therefore, it is necessary to reconfigure joint orders and develop a novel gravity compensation mechanism suitable for the new base joint.

In this paper, we aimed to develop a passive shoulder exoskeleton robot optimized for the MIS workspace. We intended to change the position of the base joint so that the singular point can be placed outside the desired workspace and develop a novel gravity compensation mechanism accordingly so that the shoulder exoskeleton robot can be used in MIS. In addition, we intended to manufacture it in a form where there is no structure between the arm and torso so that it does not cause problems when used in surgery. Finally, we plan to complete the development of the robot by configuring a test environment for performance evaluation and verifying the clinical functions of exoskeleton for deltoids with muscle assistance and movement stability through human experiments.

## 2 Fabrication of shoulder exoskeleton

### 2.1 Joint configuration and existing mechanism for shoulder assistance

The human shoulder has a complex structure, but if simply compared to a mechanical joint, it can be viewed as a ball-and-socket joint. A ball-and-socket joint is a joint in which a spherical part enters another concave cup-shaped part, enabling rotational movement with three degrees of freedom. Therefore, the shoulder joint, which follows the shape of a ball-and-socket joint, is also capable of rotating at 3° of freedom, and they include flexion/extension for lifting and lowering the arm forward/backward, abd/adduction for lifting and lowering the arm left/right, and the longitudinal axis of the arm for pro/supination. Since an exoskeleton robot that assists shoulder movements must not impede the wearer’s movements, it must have three degrees of freedom to follow all of the rotations of the previously mentioned with the assistance mechanism.

The shoulder exoskeleton robot currently developed for workers has all three degrees of freedom, and the vector of its base joint matches the direction of gravity when the wearer is in an upright posture. The direction of the arm is initially specified through rotation of the base joint, and then the wearer’s movement is assisted using the attached second joint and gravity compensation mechanism. These robots were created to be used by factory workers when performing overhead task (Maurice et al., 2019; Otten et al., 2018). When the wearer lifts the arm using the second joint in the direction pointed by the arm rotated by the base joint, the gravity compensation mechanism works in a way that reduces the load applied to the muscles by gravity (Otten et al., 2018; Renner, 2018). Then, while receiving the effect of gravity compensation, the worker can move the hand to the desired position and proceed with work by rotating about the third joint that matches the longitudinal axis of the arm. A shoulder exoskeleton robot with this mechanism appears to work well only for overhead work, but in fact, it is a structure that inevitably has limitations when the characteristics of the work change.

If we imagine at the positions of the three joints in the initial state, it can be known that the base joint and the third joint match and form a singular configuration. Therefore, the state in which a worker wearing an exoskeleton robot with this structure is standing at neutral position is a singular configuration, and the wearer may encounter various unnecessary situations. One of them is that the robot twists due to unnecessary rotation of the third joint, which may cause discomfort or large sudden rotation when going to the next posture. Additionally, as the singular configuration approaches, the determinant of Jacobian for rotation matrix approaches 0, so the force generated from the gravity compensation mechanism may cause relatively rapid rotation of the joint. In fact, all of these have a negative impact on the wearer’s work performance, but for workers performing overhead work, these positions are not a big problem when using the robot because they are outside the important workspace. However, if the workspace is different from this and the singular configuration described above is located within it, it will affect the performance of the work, and if the performance of the work is directly related to the safety of the worker, the object of the work, or humans, it can become a major problem. Since it can continue, the singular configuration must be removed or the location where it occurs must be moved.

### 2.2 New order of joints for intended workspace

In order for a shoulder exoskeleton robot with 3° of freedom to be used for tasks included in the workspace with a neutral posture, it is necessary to move or delete the singular configuration. Most work performed on a table, including MIS, inevitably involves a posture in which the upper arm is lowered from the shoulder in the direction of gravity in the workspace. Therefore, in order to eliminate the singular configuration that interferes with the smooth use of the shoulder exoskeleton robot, the configuration of the joint must be changed. Possible methods for this include twisting the joint (Christensen and Bai, 2018), adding a degree of freedom (Keemink et al., 2018), or replacing pin joint with a mechanism that operates in a sliding type (Castro et al., 2019). However, these methods are unsuitable for using the gravity compensation mechanism. The gravity compensation mechanism must always be attached vertically in the direction opposite to the ground to perform properly, therefore, when twisting the joint or increasing the degree of freedom, the direction of force cannot be assured to be opposite to gravity. In addition, in the case of a mechanism that operates in a sliding type, it is impossible to use the existing gravity compensation mechanism and a new design is required. However, it is more difficult to design because the sliding motion and its force transmission must be considered compared to the case of a simple pin joint. After it is developed and worn by the user, it is not easy to show the expected performance in all situations due to imperfections in the human-robot interface and complexity of the system. Therefore, we want to maintain the 3° of freedom but change the order of the joints to exclude the singular configuration from the MIS workspace, which is the target of the robot we want to use.

To satisfy the workspace of underhead tasks such as MIS, we changed the order of the joints so that the axis of rotation for shoulder abd/adduction is the base joint. When transmitting assistive force to the wearer using a gravity compensation mechanism, the last joint must be handled by the rotation axis of pro/supination, so that a human-robot interface can be easily constructed using a U-shaped pad. Since the shape of the human arm is similar to a cylinder, the U-shaped pad has the function of allowing the arm to contact the pad and rotate freely while receiving compensation for gravity without physically constructing the last joint for those rotation. We also wanted to use a U-shaped pad to utilize this function, so the last joint had no choice but to be fixed as the rotation axis of pro/supination, so the order of the three joints was abd/adduction, flexion/extension, and pro/supination. Since the joints are all vertical and connected in a row, if a singular configuration occurs, it will be the point where the base and the last joint overlap, and according to the order of the already determined joints, that point will be in a posture where the arms are stretched forward, and is located outside the workspace we want. However, in this case, the problem in terms of workspace can be solved by moving the singular configuration, but the previously developed gravity compensation mechanism can only be used on robots with the original base joint and sequence, so the development of a new mechanism is necessary.

### 2.3 Novel gravity compensation mechanism

A new thing to consider, as the base joint is changed to a rotation axis for abduction/adduction, is that the gravity compensation mechanism cannot be installed on the second joint. In the existing case, the direction of work is adjusted by rotating the base joint, and the gravity compensation mechanism reduces the load when lifting the second joint in the direction opposite to gravity. However, considering the changed order of the rotation axes, the gravity compensation mechanism must be affected by the rotation of the base and the second joint. Specifically, when the arm is rotated 90° about the base joint and abducted, the gravity compensation mechanism provides the best assistance force to the wearer. However, in this state, if the arm is moved in the medial direction with respect to the axis perpendicular to the ground, the arm rotates about the second joint, and the center of mass of the arm gradually approaches the center of the body; therefore, the amount of assistance provided by the gravity compensation mechanism to the abductor should gradually decrease. When the arm finally faces forward, it assumes the posture of flexion rather than abduction; thus, the assistance force for abduction should be 0. However, in this posture, from the flexion point of view, the arms are farthest from the center of the body. Hence, the assistance force provided by the gravity compensation mechanism to the muscles responsible for flexion must be maximized. For the second joint, which is responsible for the rotation of flexion/extension, the amount of assistance force received from the gravity compensation mechanism must gradually increase as the arm moves in the medial direction.

**FIGURE 1:**
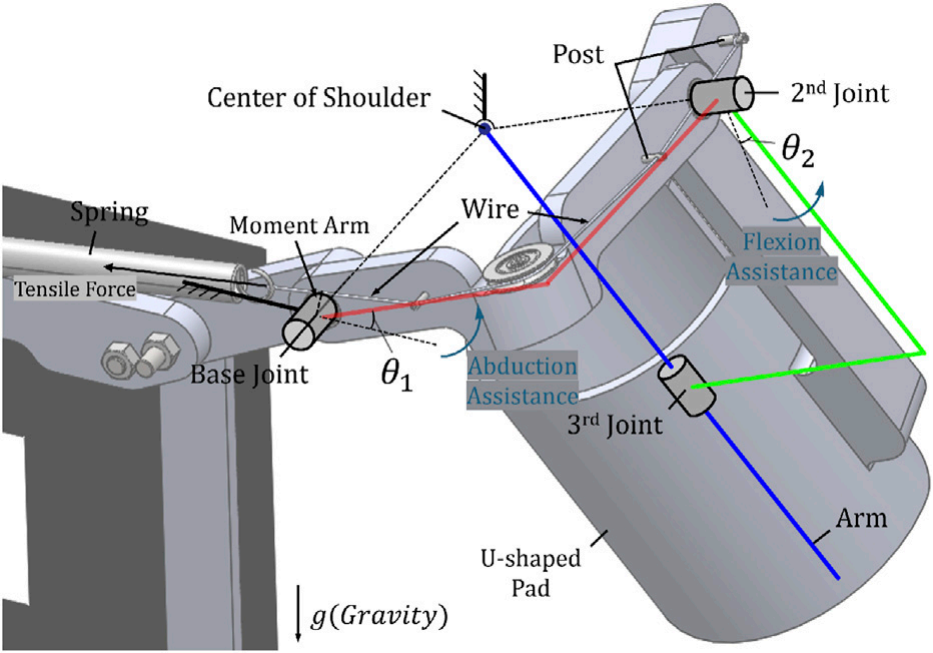
The partial view of the designed shoulder exoskeleton and the component names of system and the novel gravity compensation mechanism.

Thus, the gravity compensation mechanism to be used in accordance with the order of our newly defined joint sequence must be designed to be influenced by angle changes in the base and the second joint. We design a mechanism using a spring and a wire-roller system connected to it and a gravity compensation mechanism to couple the two joints. Figure 1 shows the three joints made. The base and the second joint are the physical axes of rotation between the links, and the third joint is a virtual joint between the U-shaped pad and the human arm for pronation/supination. The gravity compensation mechanism transmits assistance to each link by a spring and the wire post connected to it and basically adjusts the size of the assistance using the size of the moment arm, which is the distance between the vector of tensile force and the joint center. $\theta_{1}$ and $\theta_{2}$ in Figure 1 are the rotation angles of the links for the base and the second joint, respectively. In the initial state, the wire that transmits the tensile force meets the extension of the axis of the joint; thus, no moment due to the tensile force occurs. If the link rotates and $\theta_{1}$ and $\theta_{2}$ take certain values, a moment arm occurs between the extension of the joint axis and the wire; therefore, a certain moment is transmitted to the link. In addition, the two joints are coupled by a spring and a wire connected to it; thus, any value of $\theta_{1}$ and $\theta_{2}$ can affect the moment that occurs in both joints. For example, assuming that $\theta_{2}$ remains at a certain value and that there is a change in $\theta_{1}$ , the tensile length of the spring changes so that the tensile force it provides changes. As a result, the moment provided to the base joint also changes.

This mutual change in coupled joints and moments is used to solve problems caused by changes in the joint order mentioned previously. Considering the movement from abduction where $\theta_{1}$ is rotated to a certain degree to an extended state by bringing the arm in the medial direction, $\theta_{1}$ is fixed at a constant degree and continuously provides a certain moment to the muscles for abduction. During this movement, as $\theta_{2}$ increases, the tensile force provided by the spring decreases, and the assistance provided to the muscles for abduction decreases. This phenomenon is consistent with the decrease in the load of the abductor as the center of mass of the arm moves in the medial direction, making natural assistance possible. In addition, as $\theta_{2}$ increases, the size of the assistance force provided to the flexor increases proportionally, and therefore, a new gravity compensation mechanism consisting of a spring and a wire is coupled to the two joints to provide the necessary moment appropriately and naturally.

However, since the joint is coupled by a single mechanism, it provides both benefits and limitations. Specifically, the provided assistance is split by two coupled joints, and not all of their components are always in the direction opposite to that of gravity. The direction of the synthetic moment transmitted to the wearer through the U-shaped pad is not always the direction in which the arm is lifted when viewed from the center of the shoulder joint, but it inevitably has components in other directions that affect movement. Most of the provided moments are beneficial for gravity compensation, but for components that are not, it is necessary to verify whether they are acceptable limits to the effect provided by the entire system through quantitative analysis and clinical experiments. Therefore, we quantitatively calculate the degree of the beneficial gravity compensation moment and the size of the unnecessary moment in Section 3. And, we describe the clinical evaluation of the disadvantages/advantages provided to the wearer in Section 4.

### 2.4 Frames and human-robot interfaces

**FIGURE 2:**
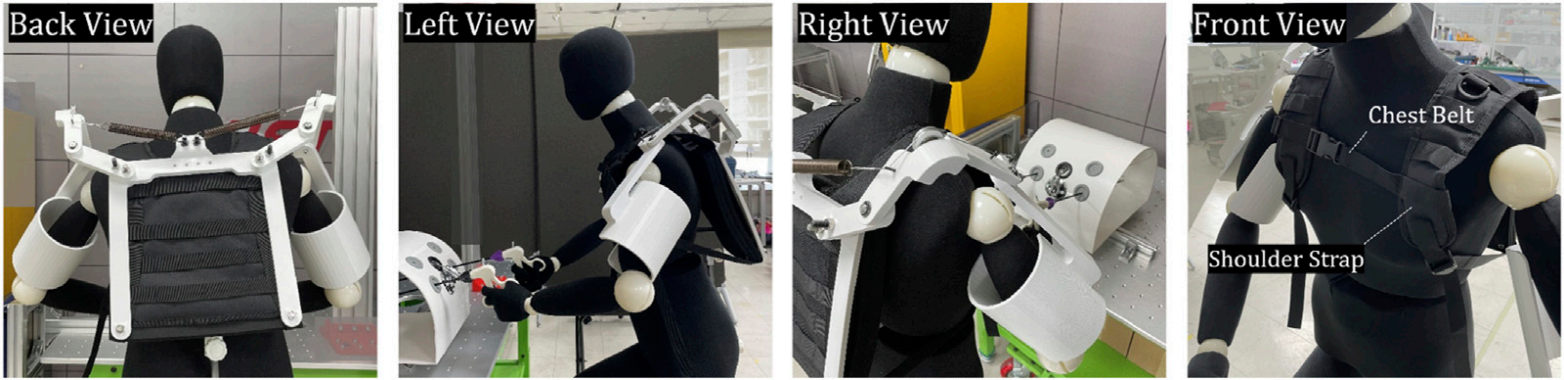
The fabricated appearance of the developed shoulder exoskeleton with mannequin captured from four directions and the included human-robot interfaces.

Appropriate–human–robot interfaces are included in the exoskeleton robot, as shown in Figure 2, to provide assistance to the wearer using the new gravity compensation mechanism mentioned in Section 2.3. As mentioned earlier, a U-shaped pad is used to provide one degree of freedom and transmit assistance to the wearer’s arm. The U-shaped pad acts as a bearing when the wearer places their arm on it, with the arm acting as a shaft. During MIS, which we consider a target, dynamic motion rarely occurs, and a specific posture is often maintained for a long time. Therefore, it is believed that the friction between the U-shaped pad and the human arm is not a major problem. Additionally, a shoulder strap and chest belt are used to attach the exoskeleton robot to the wearer. The wearer can wear the robot as a backpack using shoulder straps and adjust the belt on the chest to optimize the fit. Afterward, when they insert their arms into the U-shaped pad, the human–robot interface is completed, and they are ready to receive assistance from the gravity compensation mechanism.

## 3 Design validation

### 3.1 Workspace

By considering the range of movement of the surgeon performing MIS, the workspace of the exoskeleton robot to be developed can be determined. According to a study that observed and analyzed the intra-operative movements of surgeons performing laparoscopic surgery (Aitchison et al., 2016), which is one of the MIS, in the case of the shoulder joint we are interested in, most extreme angles are 85° for flexion, 61° for abduction during the surgery. Therefore, postures within this range should be possible in an exoskeleton robot, and the shoulder exoskeleton robot we developed can be considered to have sufficient workspace because it can be driven and assisted in those range. In addition, our shoulder exoskeleton provides assistance symmetrically to the arms for both moving back and forth, shoulder extension was not separately considered.

### 3.2 Performance of gravity compensation mechanism

#### 3.2.1 Joint torque

**FIGURE 3:**
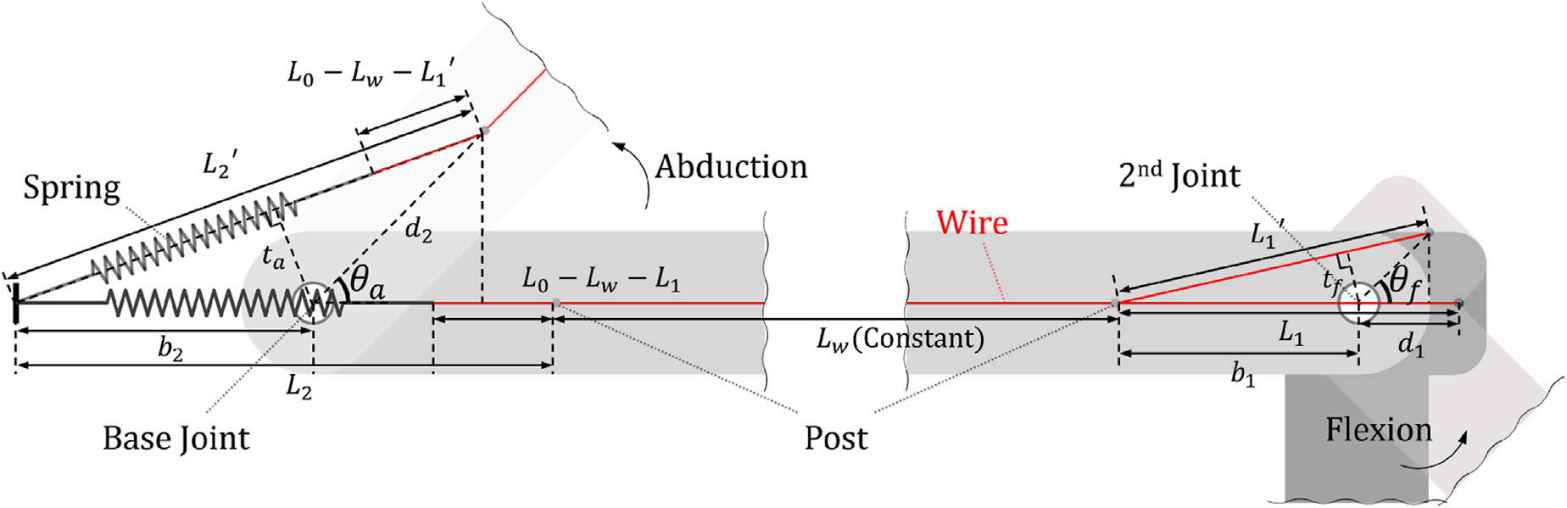
A diagram for calculating the moment of coupled two joints and the names of each element.

In order to calculate the magnitude of assistance that the gravity compensation mechanism provides to the wearer, it is necessary to first know each torque generated according to the angle change of the two rotational axes of the shoulder exoskeleton. Figure 3 shows two joints connected and coupled by a single wire. Looking at the figure, the rotation angle of abduction for the base joint is $\theta_{a}$ , and the rotation angle of flexion for the $2^{nd}$ joint is $\theta_{f}$ . As the two angles change, the length of the moment arm from the rotational axis of each joint changes, increasing the size of the joint torque. The moment arm of each joint is indicated by $t_{a}$ and $t_{f}$ , respectively, and when expressed as an equation for the rotation angle of each joint, it is the same as Equation 1.

$$t_{a}=\frac{b_{2}d_{2}\sin\theta_{a}}{L_{2}^{\prime }},t_{f}=\frac{b_{1}d_{1}\sin\theta_{f}}{L_{1}^{\prime }}$$ (1)

This change in joint angle makes the tensile length of the spring change along with the change in moment arm size, thus affecting the torque of each joint. If $x_{s}$ , which represents the tensile length of the spring, is expressed as an equation with the rotation angle of each joint, it is the same as Equation 2.

$$x_{s}=L_{2}^{\prime }-\left(L_{0}-L_{w}-L_{1}^{\prime }\right)$$ (2)

By multiplying the difference between the tensile length and the initial length of the spring by the spring constant, the current tensile force of the spring can be derived, which we named $f_{s}$ and can be expressed as Equation 3.

$$f_{s}=k\delta x=k\left(x_{s}-x_{0}\right)$$ (3)

Finally, by multiplying this tensile force $f_{s}$ by $t_{a}$ and $t_{f}$ representing each moment arm, each joint torque $\tau_{a}$ and $\tau_{f}$ could be obtained as shown in Equation 4.

$$\tau_{a}=t_{a}f_{a},\tau_{f}=t_{f}f_{s}$$ (4)

In order to determine in what direction the joint torque obtained in this way provides human-robot interaction force to the wearer, the direction of the torque must be derived.

#### 3.2.2 DH-parameters

**FIGURE 4:**
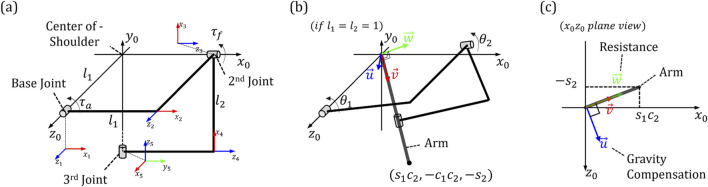
The positions and names of the coordinate systems assigned for analyzing characteristics of developed exoskeleton: **(A)** Five coordinate axes in the system **(B)** Longitudinal direction of the arm **(C)** Element vectors of assistance force for gravity compensation and resistance.

Since the direction of torque is consistent with the direction of the axis where it is generated, in order to calculate the characteristics of the torque generated at each joint, the direction of the joint axis according to the rotation angle of the joints must be known. The study of calculating the direction and coordinates of each element in a system composed of links and joints is called kinematics. By using the DH parameter developed by Denavit Hartenberg in kinematics (Craig, 2005), the rotation matrix can be easily derived and the direction of each joint can be observed. To get the DH parameters, we set a total of five coordinate axes as shown in Figure 4A. Among the three rotational joint related to shoulder rotation, the *z*-axis of the base joint was aligned with the *z*-axis of the global coordinate $\left(O_{0}X_{0}Y_{0}Z_{0}\right)$ . And then, $O_{2}X_{2}Y_{2}Z_{2}$ was assigned to the second joint, and $O_{5}X_{5}Y_{5}Z_{5}$ was assigned to the U-shaped pad for the last human-robot interface. The last coordinate axis $O_{5}X_{5}Y_{5}Z_{5}$ corresponds to the end-effector in this system, and the torque generated at the base and $2^{nd}$ joint is transmitted to the wearer through this. Therefore, the characteristics of the assistance generated vary depending on the relative rotation of $O_{5}X_{5}Y_{5}Z_{5}$ with respect to the global coordinate axis, and in order to know this, DH-parameters and a rotation matrix using them must be derived. Table 1 shows the DH-parameters for the entire system, which can be used to know the three-dimensional rotation information about global coordinates and the characteristics of the resulting joint torque. A synthetic rotation matrix can be derived using DH-parameters, and through this, the direction of each joint with respect to global coordinates can be derived. In the case of the base joint, the direction vector of torque is ${\left[0\;0\;1\right]}^{T}$ because it shares the *z*-axis with the global coordinate. And, in the case of the second joint, the direction of the rotation axis varies depending on $\theta_{1}$ , and when calculated using the rotation matrix, you can see that it becomes ${\left[\cos\theta_{1}\;\sin\theta_{1}\;0\right]}^{T}$ . In order to know the characteristics that joint torques with these directions have on the arm, it is necessary to calculate the direction vector of the arm, and the characteristics can be analyzed by comparing the joint torque and the direction vector of the arm.

**TABLE 1:** Classical DH-parameter of developed shoulder exoskeleton.

i	$\theta_{i}$	$d_{i}$	$a_{i}$	$\alpha_{i}$
1	0	$l_{1}$	0	0
2	$\theta_{1}$	0	$l_{1}$	0
3	$\frac{\pi }{2}$	$-l_{1}$	0	$\frac{\pi }{2}$
4	$\theta_{2}$	0	$-l_{2}$	0
5	$\frac{\phi }{2}$	$-l_{2}$	0	$\frac{\phi }{2}$

#### 3.2.3 Assistance characteristics

$A_{0}^{5}$ shown in Equation 5 is the rotation matrix of $O_{5}X_{5}Y_{5}Z_{5}$ with respect to the global coordinate derived using the DH parameter, and based on this, we can determine how the arm is currently positioned on the global coordinate as shown in Figure 4B and the characteristic of assistance provided by the joint torque accordingly can be analyzed.

$$A_{0}^{5}=\left[\begin{array}{cccc} s_{1}c_{1} & c_{1} & -s_{1}c_{2} & l_{1}s_{1}c_{1}\\ -c_{1}s_{2} & s_{1} & c_{1}c_{2} & -s_{2}c_{1}c_{2}\\ c_{2} & 0 & s_{2} & -l_{2}s_{2}\\ 0 & 0 & 0 & 1 \end{array}\right]$$ (5)

where $s_{i}$ and $c_{i}$ are equal to $\sin\theta_{i}$ and $\cos\theta_{i}$ , respectively. $\vec{v}$ in Figure 4B represents the longitudinal direction of the current arm, and using this direction vector and joint torque vector, we can analyze the characteristics of assistance. As shown in Figure 4C, we set direction vectors in three directions perpendicular to each other based on $\vec{v}$ for analyzing the characteristics of assistance. First, the $\vec{v}$ component of the two joint torques has no effect on the wearer. Because we used a U-shaped pad, so if this component of joint torque occurs, the U-shaped pad will only rotate the arm around the axis, making it impossible to transmit force. Therefore, when analyzing the characteristics of assistance, this component is not used and not important. Second, the effect we expect from a gravity compensation mechanism is the component of the joint torque corresponding to $\vec{u}$ , which is located on the XZ plane parallel to the ground and is perpendicular to the projection of $\vec{v}$ into that plane. Therefore, if we add each joint torque as a dot product with $\vec{u}$ , we can calculate the size of the assistance for gravity compensation. Lastly, $\vec{w}$ can be derived as an external product of $\vec{u}$ and $\vec{v}$ which is perpendicular to both, can actually be seen as a force that resists or hinders the arm from intended moving. This force can be calculated by adding the dot product of each joint torque and $\vec{w}$ . Superficially, we may think of this force as being responsible for interfering with the wearer’s task. However, if it is smaller in size than the second component, which has the effect of gravity compensation, and considering that humans use strategies to enhance joint stiffness through muscle cocontraction in tasks requiring accuracy, then it can be expected that it may be used in beneficial direction.

#### 3.2.4 Assistance validation

**FIGURE 5:**
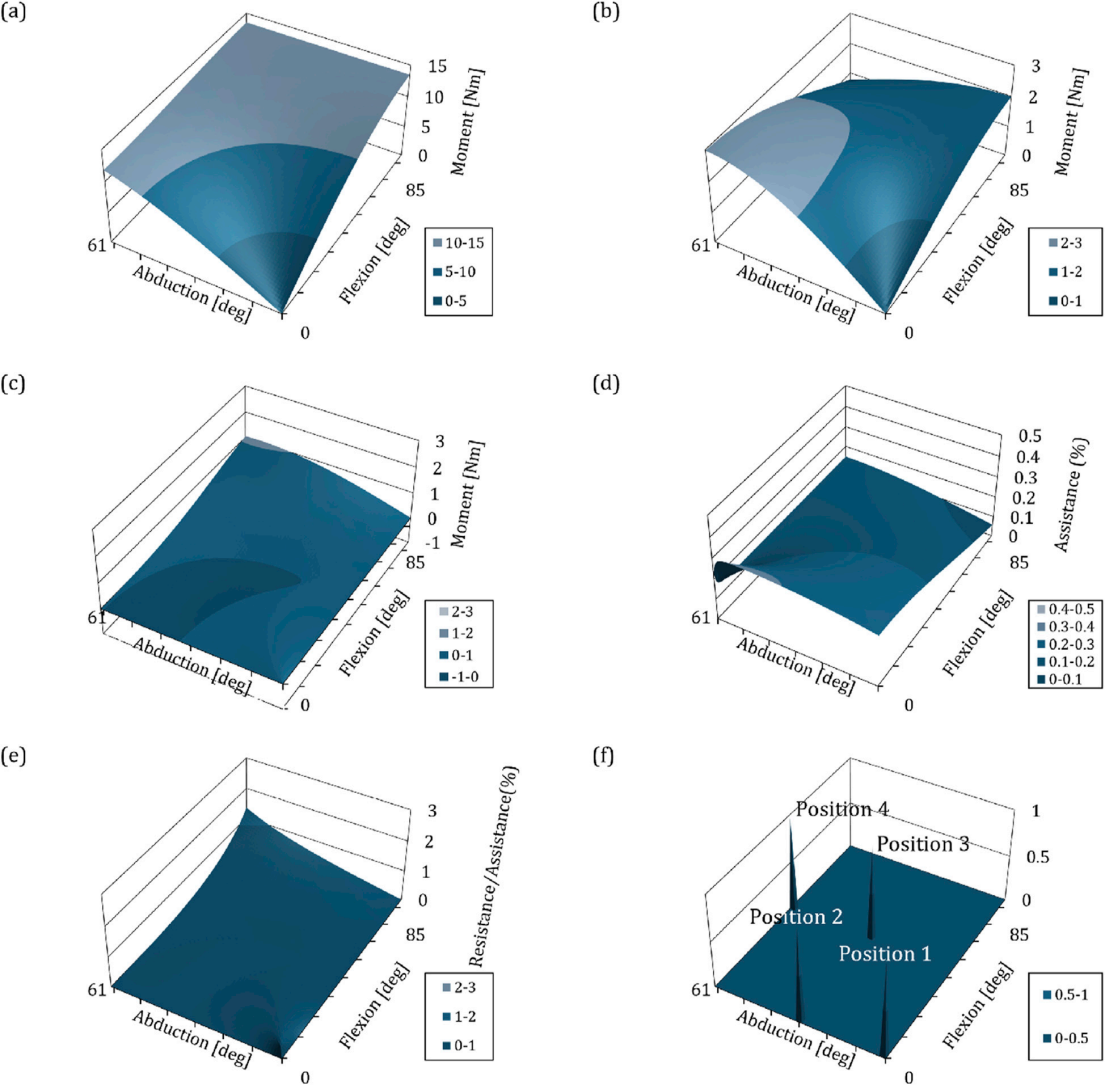
Graphs depicting the biomechanical interpretation of kinematics and mechanisms with respect to changes in angle of shoulder exoskeleton. The x and y axes, which form the base plane, represent the angles of Abduction and Flexion for the shoulder joint rotation, respectively, while the value on the z -axis represents the following: **(A)** Load of the arm on the shoulder joint due to gravity **(B)** Elements of the gravity compensation mechanism with moments opposite to gravity (Gravity compensation), **(C)** Elements of the gravity compensation mechanism with moments overlapping the direction of arm movement (Disturbance), **(D)** Ratio of assistance of the gravity compensation mechanism with respect to arm load, **(E)** Ratio between **(C)** and **(D)**, **(F)** Position of the arm posture for clinical validation to be conducted in Section 4.

To verify that the assistance characteristics calculated in Section 3.2.3 are sufficient, the load due to the weight of the arm should be calculated for each angle and then compared to the assistance at each position. Referring to research on body segment parameters (Drillis et al., 1964), for a person with height l and weight m , the length and weight of the upper arm, forearm, and hand are as shown in Table 2. Using this value, we can calculate the load added to the shoulder muscles due to the weight of the arm at each point in the workspace. We calculated the load for the maximum elbow extension position to assume the worst case scenario. In order to calculate the load, we need to know the values of l and m by specifying the human height and weight. This study is not intended for commercialization of exoskeleton robots, and therefore users with random characteristics were set up to verify performance. Even if a robot is manufactured and its performance is verified by setting a specific user, if a quantitative analysis and clinical validation of the assistance are performed in an environment that simulates MIS, which is a field of future use, the ratio can be easily adjusted according to the user’s characteristics in the future. Also, adjusting the ratio does not fundamentally change the performance of the gravity compensation mechanism and the principle of assistance provided from it. In this study, in order to verify the assistive power, an analysis of assistive characteristics was conducted targeting a specific user with a height of 180 cm and a weight of 68 kg, and Figure 5A shows the magnitude of moment affected on the shoulder due to the weight at each point of the workspace using the corresponding values.

**TABLE 2:** Average weights and lengths of body segments based on value of weight and height.

Index	Upper arm	Forearm	Hand	Whole body
Length	0.2111l	0.1731l	0.1176l	l
Weight	0.03235m	0.01813m	0.00844m	m

To verify the assistance, the ratio of the load caused by arm weight and the magnitude of assistance at a specific point must be calculated. We used the spring constant value of 1.33N/mm to derive the assistance characteristics. As mentioned in the paragraph above, the absolute value of this is not important, and although it is a value that can be changed at any time to suit the certain user, it gives us the pattern of assistance and ratio based on index of the wearer. Figures 5B,C are plotted based on the spring constant of 1.33N/mm and the assistance derived using the method in Section 3.2.3. Figure 5B shows the assistance that provides the beneficial effect of gravity compensation, and Figure 5C shows the moment related to the resistance to movement. By comparing Figures 5A,B, the assistance ratio for load of the arm weight due to gravity at each position can be derived as shown in Figure 5D. The average assistance ratio within the workspace is equal to 18.14 ( ± 5.55)% which means when constructing a shoulder exoskeleton robot using a spring constant of 1.33N/mm, it provides that size of assistance within the workspace to a wearer with characteristics of 180cm and 68 kg. In addition, Figure 5E shows the relationship between the elements that bring about the effect of gravity compensation and the elements that bring about the interference effect, with a ratio of the latter as to the size of the former. Within the workspace, the value is almost 0.3 or less, proving that the developed exoskeleton robot is effective in terms of muscle strength assistance. However, in the case of a whole workspace, a posture with a value exceeding 1 occurs. In fact, this part is close to the singular position that we wanted to exclude from the workspace, so it is rarely located during work, and because the absolute difference between the two torques is small in size (less than 1Nm), it is believed to be suitable for use by MIS surgeons. Next, based on this computational verification, we plan to conduct experiments with human subjects to verify the clinical effectiveness of the shoulder exoskeleton robot.

## 4 Clinical validation

### 4.1 Apparatus

**FIGURE 6:**
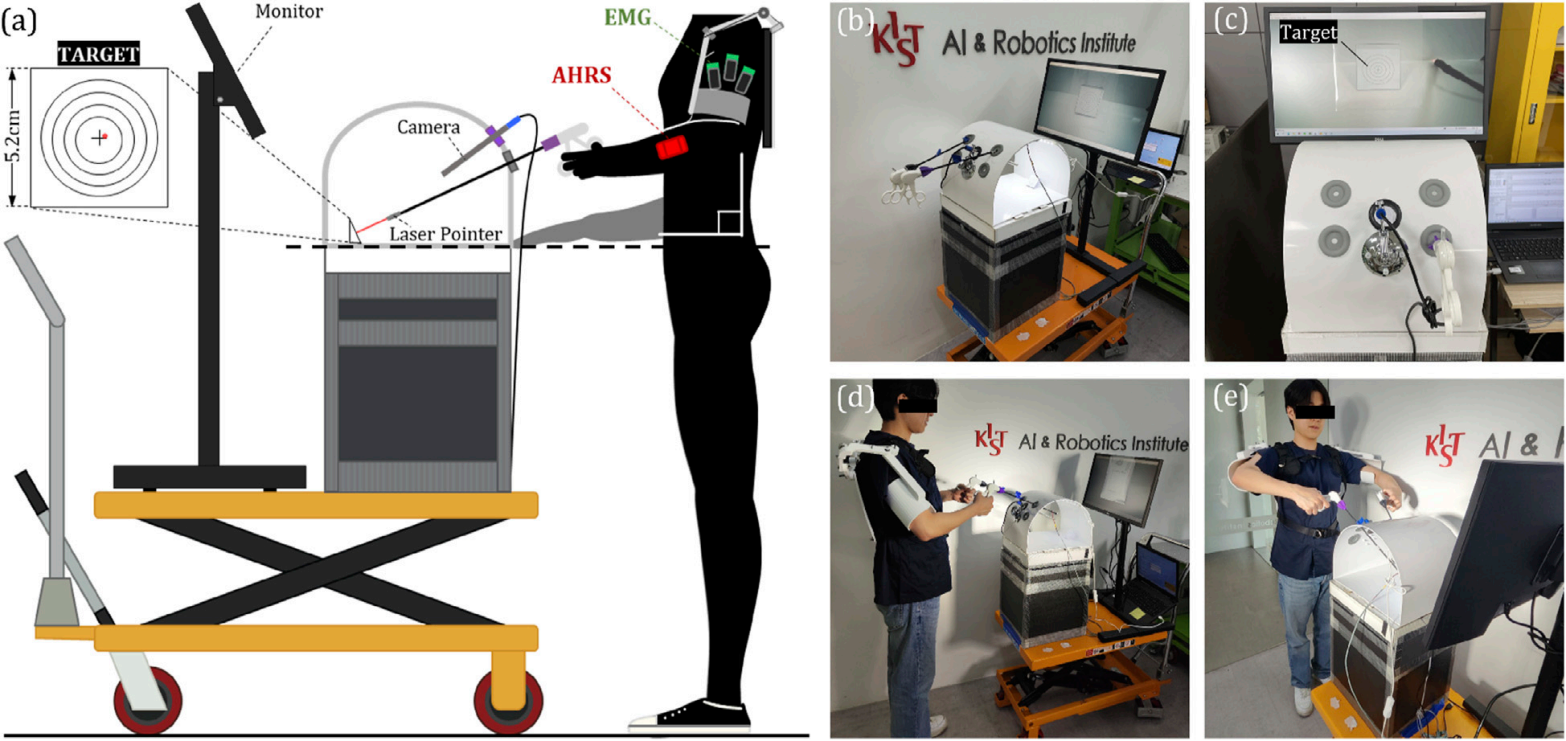
**(A)** A diagram illustrating the apparatus developed for clinical validation and its components, **(B)** A photo of the actual fabricated apparatus, **(C)** A view of the screen from the perspective of the experimental participants, **(D)** A side view of the participant wearing the exoskeleton and participating in the experiment, **(E)** A frontal view of the participant.

All systems must be verified for performance in actual use environments or situations that simulate it, and the novel gravity compensation mechanism developed in this study and the shoulder exoskeleton robot are specific to the user performing MIS, so it should be examined whether enough clinical performance is provided to the wearers in similar situations with MIS. Therefore, we evaluated the clinical performance of our shoulder exoskeleton using a kit that can train laparoscopic surgery, which is one of MIS (Crespin et al., 2018). The kit was developed to help trainees practice in situations such as laparoscopic surgery, and is designed in the form of a box that simulates the target area with holes for inserting cameras and surgical tools. This kit can be used to practice surgery using surgical tools by placing a target object in a box, viewing the screen recorded from a camera inserted through the hole in real time, and performing a given task. To evaluate the clinical performance of the developed system, we installed a target in a box as shown in Figures 6A,B, and also attached a laser pointer to the end of the surgical tool so that subjects wearing a shoulder exoskeleton robot could look at the screen like Figure 6C and use the surgical tool to perform given tasks. The kit was used by giving the task of pointing the laser that comes out to the exact center of the target.

### 4.2 Subjects and protocols

**FIGURE 7:**
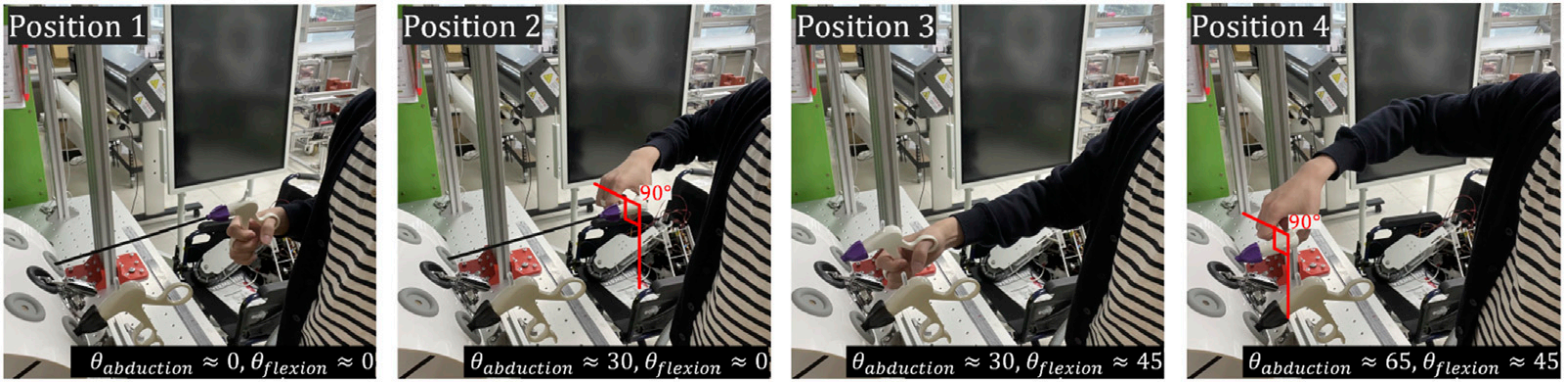
Photos of four postures to be used for clinical validation. The positions for these postures are depicted in Figure 5F.

A total of 10 subjects participated in the experiment, and they were recruited without restrictions on height, weight, or gender. The subject visited the laboratory for 1 day and performed the given tasks according to the given protocol for cases wearing and not wearing the exoskeleton robot. The protocol was about four postures that can be taken while performing MIS, and the postures are shown in Figures 6D,E, 7. Position 1 is a posture in which the surgical tool is inserted into the kit and held parallel to the ground, Position 2 is a posture in which only the surgical tool is rotated and held perpendicular to the ground in the same state as Position 1, and Position 3 and Position 4 are positions which only the tool is inserted more deeply inside the box than Position 1 and 2, respectively. One set consists of maintaining from positions 1 to 4 in order, for 30 s each for a total of 2 min. The subject repeated a total of 5 sets, with a 2-min break between sets. When participants performed a given protocol, they wore the exoskeleton robot once and did not wear it once, and the order was randomly determined. Table 3 contains a summary of the experimental protocol conducted during one visit, which was approved by the Institutional Review Board of the Korea Institute of Science and Technology. (IRB number: KIST-202304-HR-004).

**TABLE 3:** The sequence of events when participants visit the laboratory to conduct the experiment.

Order	Time (min)	Task
1	10 ∼ 15	Oral explanation of experiment details and signing of consent form
2	5	Attaching AHRS and surface electromyography sensors
3	5	Performing maximum voluntary contraction
4	5	Trial 1: Position 1 ∼ Position 4 (30 s × 4 position)
5	5	Rest
6	5	Trial 2
7	5	Rest
8	5	Trial 3
9	5	Rest
10	5	Trial 4
11	5	Rest
12	5	Trial 5

### 4.3 Measurements

#### 4.3.1 Muscle activation

Because the primary purpose of using the shoulder exoskeleton robot is to reduce the user’s muscle load and fatigue through gravity compensation, we measured the participants’ shoulder muscle activation during the clinical experiment. The EMG (Electromyography) sensor of the Delsys trigno system was attached to the participant’s shoulder muscles which are deltoid anterior, medial, and posterior, as shown in Figure 6A, and the activity of the three muscles was measured. In the protocol, maximum voluntary contraction (MVC) was performed to normalize the value coming from the EMG sensor before executing the task, and the EMG value measured during the experiment was converted to RMS according to a general processing technique and converted into a ratio (%) to MVC for analyzing.

#### 4.3.2 Movement variability

The clinical effect achieved by providing muscle assistance to the wearer using a shoulder exoskeleton robot is to reduce the muscle strength, but it is also intended to increase the accuracy of hand work by enhancing arm stability. Therefore, we measured the degree of arm variability by measuring the 3-axis acceleration value of the arm using the AHRS (Attitude and Heading Reference System) sensor. The AHRS sensor is mounted on a strap so that experiment participants can wear it on their arms, as shown in Figure 6A. Among the measured 3-axis acceleration values from AHRS sensor, excluding those in the longitudinal direction of the arm, only the remaining 2-axis directions were used. After scatter plotting the two-axis acceleration values measured during each position, covariation was calculated to derive the principal axis and standard deviation of the corresponding direction (Choi and Baek, 2020). The RMS value of the two derived standard deviations was used as a value representing the movement variability during the relevant period.

#### 4.3.3 Data acquistion

The data acquired in this experiment include three-axis acceleration values measured by the AHRS sensor which are attached to the upper arm, and EMG values of three deltoids measured by the Delsys trigno system. Acceleration values were acquired using STM32F407-Disc1 development board of STMicroelectronics, EMG values were acquired using Delsys’ own software, and data from two systems was synchronized using the trigger module provided by the Delsys. The acceleration value was measured at 500 Hz, and the EMG value was measured at 1,259 Hz, and a bandpass filter of 20 400 Hz was applied (Konrad, 2005), and it was converted to RMS based on a window size of 1.25s and used to calculate MVC (%).

#### 4.3.4 Statistical analysis

We used MATLAB for data analysis, which was used to derive the indices mentioned in Sections 4.3.1 and Section 4.3.2. First, the experimental data conducted according to the protocol in Section 3.2 was divided into datasets for each position. Therefore, 8 datasets were generated per experiment participant depending on whether the robot was worn or not, and a total of four positions. Position 1 is the posture in which the surgical tool is inserted into the kit and held parallel to the ground. It is the most basic and requires minimal muscle activation, and since the posture without wearing the exoskeleton robot is common, it was decided to use that position 1 as the standard posture. Therefore, the 8 datasets were converted into ratios to the data at position1, and after normalization was performed, the data of a total of 10 people could be grouped into one. As a result, the set was repeated 5 times per participant, and because the number of participants was 10, it was possible to construct a dataset containing a total of 50 data per case of one indicator. To compare these groups, we plotted eight data sets using a box-and-whisker graph. In addition, after identifying and removing outliers using the interquartile range, one way-ANOVA was used to derive positions where significant differences were observed in muscle activity and movement variability according to wearing the exoskeleton robot.

### 4.4 Experimental results

#### 4.4.1 Muscle activation

**FIGURE 8:**
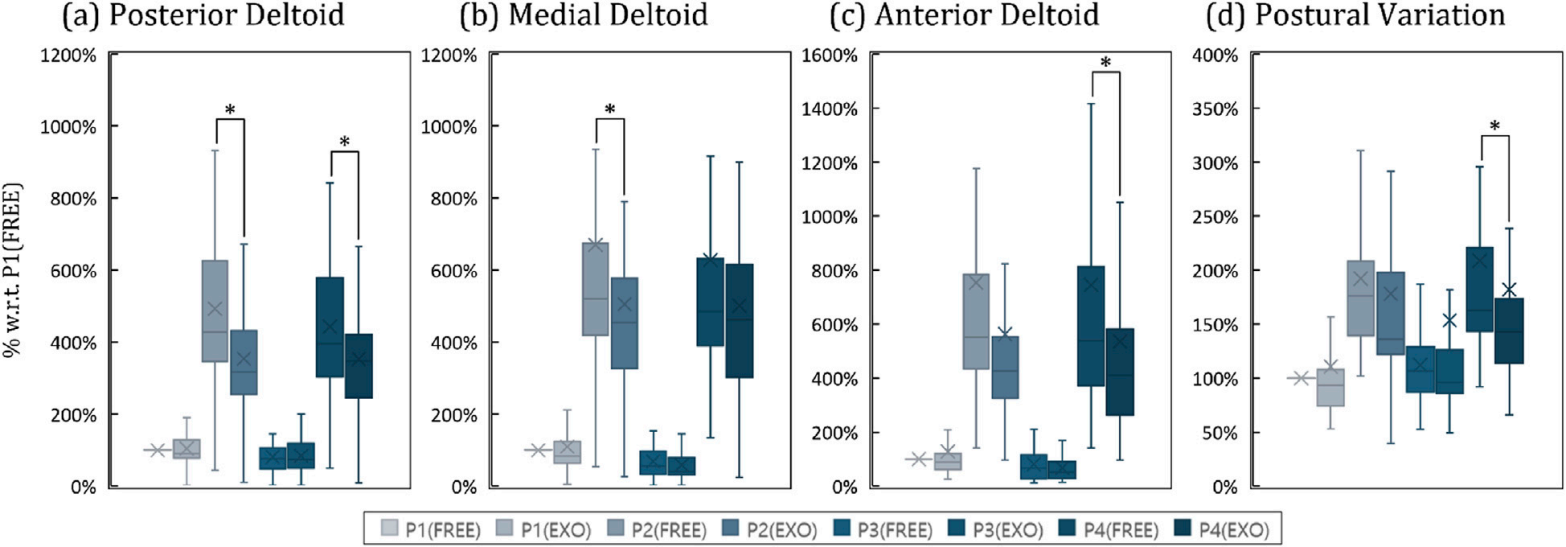
Box-and-whisker plots representing the results of the clinical experiment. Pn denotes the results for the *n*th posture. FREE and EXO indicates the results without and with shoulder exoskeleton. **(A)** Activation of the posterior deltoid by case, **(B)** Activation of the medial deltoid by case, **(C)** Activation of the anterior deltoid by case, **(D)** Postural variation by case.

Figures 8A-C is a box-and-whisker graph showing the degree of muscle activation (ratio to position1 and free) of three deltoids depending on whether or not the exoskeleton robot is worn. And Table 4 shows the average value of muscle activations for each data set excluding outliers. The average value of muscle activation was lower when the exoskeleton robot was worn for each position of all three deltoids than when the exoskeleton robot was not worn, indicating that the exoskeleton robot had beneficial effects. In addition, as a result of one-way ANOVA, significant differences were observed at positions 2 and 4 for the deltoid posterior, at position 2 for the deltoid medial, and at position 4 for the deltoid anterior, depending on the wearing of the exoskeleton robot. Table 4 shows the rate of decrease in muscle activity due to wearing the exoskeleton robot for the four positions. For the positions where significant differences were observed, the exoskeleton robot resulted in an average decrease in muscle activity of 23.37%.

**TABLE 4:** A table quantitatively calculated from the results of the clinical experiment. P n denotes the results for the $n^{th}$ posture. FREE and EXO indicates the results without and with shoulder exoskeleton. Each result is expressed as a proportion relative to P1(FREE), and the proportional changes between FREE and EXO are also indicated.

Index	P1 (Free)	P1 (EXO)	P2 (Free)	P2 (EXO)	P3 (Free)	P3 (EXO)	P4 (Free)	P4 (EXO)
Posterior	1.000	1.076	4.811	3.489	0.796	0.876	4.465	3.421
Deltoid	(+7.6%)		(−27.5%)*		(+10%)		(−23.4%)*	
Medial	1.000	0.929	5.775	4.819	0.659	0.511	5.375	4.735
Deltoid	(−7.1%)		(−16.6%)*		(−22.6%)		(−11.9%)	
Anterior	1.000	0.896	6.043	4.568	0.747	0.576	6.140	4.538
Deltoid	(−10.5%)		(−24.4%)		(−23.0%)		(−26.1%)*	
Movement	1.000	0.954	1.793	1.574	1.083	1.045	1.740	1.400
Variability	(−4.6%)		(−12.2%)		(−3.1%)		(−19.5%)*	

#### 4.4.2 Movement variability

Figure 8D is a box-and-whisker graph showing movement variability according to position and whether or not an exoskeleton robot is worn. And Table 4 shows the average movement variability of the groups excluding outliers for the eight data sets. Looking at this, it can be seen that the movement variability values were lower when the exoskeleton robot was worn in all four positions than when it was not worn, indicating that the movement was stable. In addition, as a result of one way-ANOVA, it was observed that in the case of position 4, a statistically significant difference occurred with respect to whether or not an exoskeleton robot was worn. Table 4 shows that movement variability decreased by 9.85% on average when wearing an exoskeleton robot for four positions, and in the case of position 4, where a significant difference was observed, the exoskeleton robot provided movement stabilization of 19.51%.

## 5 Discussion

Underhead tasks such as MIS require significant muscle activation due to inappropriate working environments, use of unergonomic tools, long working hours, or high precision, even though the workspace is below the head. We wanted to develop a shoulder exoskeleton robot that could reduce the load on shoulder muscles of workers who engage in those tasks. Accordingly, we changed the joint order of the exoskeleton robot to adapt focused workspace, developed a novel gravity compensation mechanism, and attempted to verify its performance through mathematical calculations and clinical trials on human subjects. In this section, we would like to discuss 1) the novelty of the developed shoulder exoskeleton robot, 2) reliabilities on computational verification methods, and 3) opinions on clinical validation results.

First, we developed a new gravity compensation mechanism to modify the workspace of the shoulder exoskeleton robot to suit the target for which it will be used. We moved the location of the singularity, changed the order of the joints so that the robot could be used in the changed workspace. And we also developed a new gravity compensation mechanism that could replace the existing one that could not be used because of change in order of joints. In fact, it is a self-evident fact that tasks with an overhead workspace place greater muscle load on the shoulders because the hands have to be raised above the head. And existing robots have been created for that purpose and are mostly used on assembly or production lines in factories. However, muscle fatigue is not only affected by the size of the activation level, but also the time the activation level is maintained (Enoka, 1995). Among tasks with an underhead workspace, there are some occasion that require maintaining a specific posture for a long period of time, such as MIS. In this case, even if the hands and arms are below the head, the time they are maintained is long, which inevitably increases fatigue in the shoulder muscles so that the shoulder exoskeleton robots can be used even for workers performing these tasks. Additionally, in the case of underhead work, accuracy at the fingertips is often required, and in order to achieve this, joint stiffness must be increased through cocontractions of the muscles related to wrist, elbow, and shoulder joints, which causes accelerated muscle fatigue (Hunter et al., 2002). We confirmed this fact through previous research and proved that shoulder muscle fatigue worsens proprioception and ultimately has a negative effect on fingertip accuracy (Choi and In, 2022). In this perspective, underhead work requires muscle assistance as much as overhead work, and fatigue in work requiring accuracy is directly related to work performance. Therefore, the gravity compensation mechanism and shoulder exoskeleton robot for underhead-workspace are said to have sufficient novelty.

Second, we would like to describe whether the computational verification of the performance of the developed gravity compensation mechanism was correct and sufficient. Through kinematic analysis, we plotted the assistance according to the rotation angle of the exoskeleton robot joints and verified the computational performance by comparing it with the load due to gravity of the arm. We proceeded with this process by specifying the user’s body characteristics and spring constant values. As mentioned in Section 3.2.4, these factors are not very important in verifying the computational assistance. Humans have body structures of different sizes and weights, and it takes too much time and cost to custom-make them considering the wearer. Therefore, it is most effective to manufacture an exoskeleton robot using the ratio of average human body parameters, and we also conducted computational verification using such ratio in Section 3.2.4. Additionally, when changing the spring constant, the torque generated according to the rotation angle of each joint will change in proportion to its change rate. Therefore, the specific user’s body characteristics and spring constants that we used for computational verification all affect the assistance at a certain rate. The load on the shoulder due to the arm’s gravity derived using body characteristics and the assistance of exoskeleton is considered the assistive performance of the exoskeleton robot. And this is related to the ratio, so even if the user changes them the fundamental assistive characteristics will not change, only the ratio will change. In fact, we only wanted to verify whether the developed gravity compensation mechanism operates properly and whether the direction of the force provided is correct, and we are not curious about what assistance ratio provides good clinical performance to the wearer. Therefore, these methods have sufficient performance and it can be seen that it contributed to enough verification.

Lastly, we would like to discuss the results of an experiment conducted to clinically verify the reduction of muscle activity and stabilization of movement in the shoulders of workers performing the underhead task, which was our ultimate goal. We achieved an average reduction in muscle activity of 14.61% and stabilization of movement of 9.85% through human experiments in an environment similar to MIS, which was our main target. In fact, in the case of position 1 and position 3, it is difficult to discuss performance because the load on the shoulder is smaller than the other two, and in fact, the decrease in muscle activation was noticeably greater in positions 2 and 4. In addition, a statistically significant decrease in the muscle activation of the three deltoids by wearing the exoskeleton robot was observed only in those two positions. In addition, the decrease in movement variability was observed to be greater in position 2 and 4 than in the other two cases and was statistically significant in position 4. These clinical results fully demonstrate the performance of the developed gravity compensation mechanism. Through computational verification, we claimed that developed shoulder exoskeleton robot would be able to provide the wearer with an average reduction in muscle activity of 16.26% within the entire workspace, and this value was found to be similar to the clinical value of 14.61%. In our computational verification, we limited the target to humans with the characteristics of 180 cm and 68 kg, but we did not limit physical characteristics when recruiting experimental participants. Therefore, it is assumed that some errors may have occurred due to the different weight and size of each person. To summarize, our developed exoskeleton can reduce deltoid muscle load by about 14.61% and decreased arm tremor by about 9.85% for a random user within the MIS workspace (Maximum flexion: 85°, Maximum abduction 61°) specified in Section 3.1. And these values are not fixed and may vary depending on the stiffness of the spring used for gravity compensation mechanism.

In addition, we attempted to verify the effect on the wearer of forces from gravity compensation mechanism that hinder or resist movement through clinical experiments. First, when wearing the exoskeleton robot, muscle activation and movement variability decreased in all positions, so it was found that the interfering force was always smaller than the beneficial force, thus proving its utility as an exoskeleton robot. In fact, since the deltoid medial is only used to lift the arm, the muscles that can be affected by the interfering force are the deltoid anterior, which moves the arm forward in plane, and the deltoid posterior, which moves the arm backward (Liu et al., 1997). However, in addition to these functions, these two muscles also perform the same role of lifting the arm, like the deltoid medial. Therefore, the activation used when lifting the arm is reduced by the gravity compensation mechanism, and this effect is larger than the amount of activation that must be increased due to the interfering force. As a result, it can be assumed that muscle activation tends to decrease when wearing the exoskeleton robot.

Another function of the two muscles is that although they have opposite functions, they can improve the stiffness of the joint through cocontraction (Missenard et al., 2008). The task given to the participants in this study was to keep the laser coming from the tip of the tool pointed at the exact center of the target, which required fingertip precision. Normally, humans adopt a strategy of improving joint stiffness by increasing the cocontraction of arm muscles to improve fingertip accuracy, and the participants in our experiment probably used the same strategy. Therefore, the deltoid anterior and posterior muscles would have more activated than just for arm lifting. At this time, the interfering force has only one direction and would have affected only one of the deltoid anterior and posterior. Therefore, in a certain position, either the deltoid anterior or posterior may have used this interfering force to compensate for some of the activation used to improve joint stiffness, and as a result, the deltoid in that direction can be expected to have decreased activation. If so, this force can be thought of as a beneficial force that improves joint stiffness rather than an impediment. For example, in the case of position 4, where the interfering force is the greatest, its size is positive so that it would have had the effect of reducing the muscle activation of deltoid anterior which took charge of improving joint stiffness. This can be additionally explained by quantitative values. Even though this posture was the most difficult, it was found that the deltoid anterior muscle activity decreased the most when wearing the exoskeleton robot compared to the other three postures. And, the only movement variability in this position was statistically significantly reduced by wearing the exoskeleton robot. Therefore we concluded that the adverse effect of the interfering force had a smaller effect on muscle activation than the beneficial effect of the gravity compensation force. And we also considered that the interfering force can have a beneficial effect that helps joint stiffness. So, it can be argued that our shoulder exoskeleton robot has sufficient utility to reduce the wearer’s muscle activation and also improve the stability of movement.

Our study has several limitations, which led us to establish new plans for future work. First, rather than using the customized size and spring constant of the exoskeleton robot to the wearer, one specific piece of hardware was used on a random experiment participant. Nevertheless, since the exoskeleton robot reduces the wearer’s muscle activation and improves the stability of movement, there is enough possibility for clinical results to be improved if these values are optimally revised considering the wearer’s body. Additionally, while we developed a robot to assist arm movements, we did not consider the shoulder girdle. This decision was made because it was important to focus on the dominant movement in our research, and the developed robot uses a U-shaped pad instead of a rigid human-robot interface, allowing us to exclude this aspect. However, in future research and clinical trials, we plan to examine this area and take any necessary measures if required. Second, because the tasks we assigned to the participants were to verify the basic performance of the exoskeleton robot, only muscle activation and movement stability were measured, which were insufficient for in-depth analysis. For example, our designed task operating time was too short to take into account muscle fatigue and therefore could not be shown to performance of exoskeleton in situations where fatigue can be generated. And it can be assumed that because the stability of the arm was improved, the accuracy of the fingertips was also guaranteed, but it was not possible to prove this with quantitative values or results. And because we focused on MIS, we conducted experiments on static postures, but performance cannot be guaranteed for tasks that require both fingertip accuracy and large portion of dynamic postures. Of course, studies have shown that during 50%–80% of the time in MIS (minimally invasive surgery) (Szeto et al., 2012), the shoulder joint remains in a static position with a movement speed of less than 1° per second. Holding the shoulder joint in abduction or flexion for extended periods increases muscle fatigue proportionally to the angle held and the duration (Chaffin, 1973; Kaur and Mishra, 2008). However, we did not quantitatively measure this using the robot we developed. Additionally, as the remaining 20%–50% involves dynamic movements, verification for this aspect is also necessary. Therefore, we will create an experimental device that could quantitatively measure the accuracy of the fingertips, give participants a task combining static and dynamic posture, and then conduct a clinical experiment by increasing the task time to allow fatigue to accumulate. Third, we did not quantitatively analyze the impact of the robot’s own weight on other joints and muscles. The robot we developed does not require actuators or a battery; it provides assistive force through springs and mechanisms, and its frame is 3D printed, making it very lightweight. Therefore, we did not believe that the weight of the robot would have a significant adverse effect and focused on the reduction of arm tremors that could be observed regardless of weight considerations. However, this issue is an important factor in exoskeleton robot development, and we plan to evaluate it quantitatively through further experiments and analysis. Lastly, we will find the optimal spring constant through simulation or clinical experiments so that it can be possible of conducting in-depth physiological analysis and finally we can improve the completeness of the exoskeleton robot with novel gravity compensation mechanism.

## Data Availability

The raw data supporting the conclusions of this article will be made available by the authors, without undue reservation.
